# Osteoarthritis patients exhibit an autonomic dysfunction with indirect sympathetic dominance

**DOI:** 10.1186/s12967-024-05258-9

**Published:** 2024-05-16

**Authors:** Rebecca Sohn, Tina Assar, Isabelle Kaufhold, Marco Brenneis, Sebastian Braun, Marius Junker, Frank Zaucke, Georg Pongratz, Zsuzsa Jenei-Lanzl

**Affiliations:** 1 Dr. Rolf M. Schwiete Research Unit for Osteoarthritis, Department of Trauma Surgery and Orthopedics, Goethe University Frankfurt, University Hospital, Marienburgstr. 2, 60528 Frankfurt Am Main, Germany; 2 Faculty of Biological Sciences, Goethe University Frankfurt, 60438 Frankfurt, Germany; 3 Department of Trauma Surgery and Orthopedics, Goethe University Frankfurt, University Hospital, 60528 Frankfurt, Germany; 4 Department of Orthopedics, Tabea Hospital Hamburg, 22587 Hamburg, Germany; 5 Division of Rheumatology and Clinical Immunology, St. John of God Hospital, Regensburg, Germany; 6 Medical Faculty, University of Regensburg, 93053 Regensburg, Germany

**Keywords:** Osteoarthritis, Autonomic dysfunction, Sympathetic activity, Parasympathetic activity, Heart rate variability, Chronic stress, Pain

## Abstract

**Background:**

Osteoarthritis (OA) is a chronic degenerative joint disease causing limited mobility and pain, with no curative treatment available. Recent in vivo studies suggested autonomic alterations during OA progression in patients, yet clinical evidence is scarce. Therefore, autonomic tone was analyzed in OA patients via heart rate variability (HRV) measurements.

**Methods:**

Time-domain (SDRR, RMSSD, pRR50) and frequency-domain (LF, HF, LF/HF) HRV indices were determined to quantify sympathetic and parasympathetic activities. In addition, perceived stress, WOMAC pain as well as serum catecholamines, cortisol and dehydroepiandrosterone-sulphate (DHEA-S) were analyzed. The impact of the grade of disease (GoD) was evaluated by linear regression analysis and correlations with clinical data were performed.

**Results:**

GoD significantly impacted the autonomic tone in OA patients. All time-domain parameters reflected slightly decreased HRV in early OA patients and significantly reduced HRV in late OA patients. Moreover, frequency-domain analysis revealed decreased HF and LF power in all OA patients, reflecting diminished parasympathetic and sympathetic activities. However, LF/HF ratio was significantly higher in early OA patients compared to late OA patients and implied a clear sympathetic dominance. Furthermore, OA patients perceived significantly higher chronic stress and WOMAC pain levels compared to healthy controls. Serum cortisol and cortisol/DHEA-S ratio significantly increased with GoD and positively correlated with WOMAC pain. In contrast, serum catecholamines only trended to increase with GoD and pain level.

**Conclusions:**

This prospective study provides compelling evidence of an autonomic dysfunction with indirect sympathetic dominance in early and late knee OA patients for the first time based on HRV analyses and further confirmed by serum stress hormone measurements. Increased sympathetic activity and chronic low-grade inflammation in OA as well as in its major comorbidities reinforce each other and might therefore create a vicious cycle. The observed autonomic alterations coupled with increased stress and pain levels highlight the potential of HRV as a prognostic marker. In addition, modulation of autonomic activity represents an attractive future therapeutic option.

**Graphical abstract:**

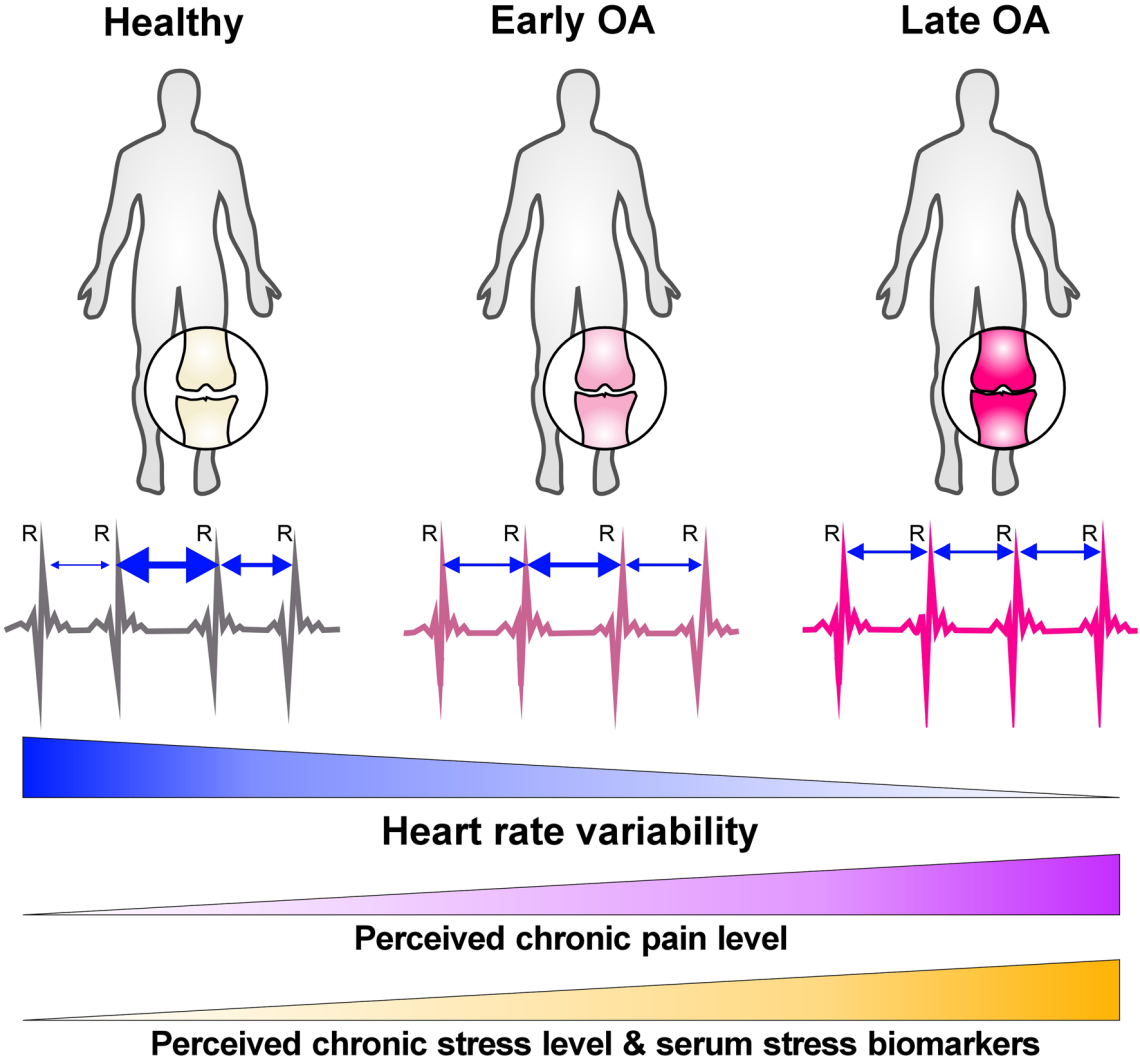

**Supplementary Information:**

The online version contains supplementary material available at 10.1186/s12967-024-05258-9.

## Background

Osteoarthritis (OA) is a multifactorial chronic degenerative joint disease characterized by a wide range of anatomical and physiological changes [1, 2]. Even though OA can affect any synovial joint, it occurs most often in the knee regarding larger joints [3]. In OA, progressive degeneration of articular cartilage, subchondral sclerosis and synovial inflammation form a vicious circle that manifests in chronic pain, reduced mobility and the onset of chronic comorbidities [4]. Pharmacological treatments for OA are mostly restricted to symptom relief and to date, there is no disease-modifying drug available counteracting OA progression [5]. Therefore, in many cases, total knee arthroplasty (TKA) remains as the only final therapeutic intervention for late OA. Since over 590 million people worldwide suffer from OA, it is not surprising that the management of this disease is associated with an enormous socioeconomic burden [6]. A major stumbling block in treating clinical OA has been the inability to identify all its risk factors and to detect its early onset. OA has a variety of risk factors which increase the individual susceptibility to the disease (e.g. age, female sex, joint biomechanics, genetic predisposition and body weight) or alter the biomechanical stability of individual joints (e.g. injury, repetitive joint use and joint malalignment) [7].

Recently, a possible autonomic nervous system (ANS) dysfunction with a shift towards a more prominent sympathetic nervous system (SNS), gained prominence in OA research as an additional risk factor [4]. The ANS plays a critical role in joint homeostasis and is implicated in the pathogenesis of OA [8–10]. Next to the enteric nervous system, the ANS comprises the SNS and the parasympathetic nervous system (PNS), which often exert antagonistic effects. The SNS is activated in terms of stress and prepares the body for coherent and coordinated action by an increased heart rate and stress hormone production. In response to stress stimuli, the SNS and the hypothalamic–pituitary–adrenal (HPA) axis become activated. While activation of the SNS results in catecholamine release from peripheral nerve endings as well as adrenal medulla [11], elevated HPA axis activity leads to increased cortisol production in the adrenal cortex [12]. Both stress axes increase the body’s metabolic rate to provide energy supply. The concentrations as well as the ratio of cortisol and its counterpart dehydroepiandrosterone sulfate (DHEA-S) provide an indicator for catabolic/anabolic balance and chronic stress [13]. Once the stressful condition is over, the PNS decreases heart rate and returns the body to homeostasis in a state of rest and digest [7]. In a healthy environment, sympathetic and parasympathetic effects are tightly coordinated and balanced, providing homeostasis for various physiological functions such as heartbeat, blood pressure, respiration, digestion, temperature regulation, and sexual arousal [14, 15]. Moreover, the ANS is a pivotal regulatory mediator between the brain and the immune system, with SNS activity having a particularly profound influence on inflammatory processes [9]. Thus, an altered autonomic balance could increase vulnerability to inflammatory stimuli or impair the ability to combat inflammatory pathologies, for example during OA [4].

The heart rate variability (HRV) is a suitable measure to analyze the (im)balance of SNS and PNS activities [16]. HRV describes the fluctuation in the time intervals between adjacent heartbeats [17], with high variation between heartbeats in parasympathetic mode and low variation in sympathetic mode [7]. The PNS primarily determines HRV, with more PNS activity resulting in higher HRV [18]. By keeping the HRV in an ideal range, the PNS and SNS ensure that the body can adapt quickly to the physical and psychological challenges of maintaining homeostasis [19]. HRV measurement is a well-established method for assessing ANS activity and has been shown to be clinically relevant [20]. It is also suitable for use in a range of clinical populations because it is non-invasive, fast, and easy-handling. Various pathologies may involve either a loss or an increase in this complexity and, particularly a loss of variability, indicated by reduced HRV, signals an impaired system and is strongly associated with an increased risk of mortality [17]. Since decreased HRV is also related to several other diseases, it is worldwide used as an indicator of health status [21]. For example, in RA, impaired vagus nerve function dampens the parasympathetic tone which is reflected by decreased HRV [22, 23] and accompanied by an increased inflammatory status [18].

Preliminary data from animal studies suggest a potential shift of the autonomic nervous system measured via heart rate response variables also in OA [24, 25]. However, to date there is no data available investigating the autonomic tone in OA patients or autonomic alterations and their association with clinical symptoms such as pain in different stages of the disease.

The aim of this study was to examine a potential autonomic shift during OA progression. Therefore, we assessed the HRV in healthy controls as well as in OA patients at an early and a late stage of the disease. In addition, perceived stress as well as pain levels were examined and associations with further stress-related factors such as serum catecholamines and cortisol were investigated. This study will therefore contribute to a better understanding of OA etiology and might help to develop novel treatment strategies.

## Patients and methods

### Data source and study population

This prospective monocentric study (DRKS-ID DRKS00018876) was conducted at the University Hospital Frankfurt, Department of Trauma Surgery and Orthopedics and approved by the Ethics Committee of the Medical Faculty of the Goethe University Frankfurt am Main, Germany (vote Nr.: 19-347) in compliance with the Helsinki Declaration. Patients and/or the public were not involved in the design, or conduct, or reporting, or dissemination plans of this research. Study participants were recruited between July 2020 and February 2023. Healthy probands were acquired using an approved study flyer. All subjects were informed about the study in writing prior to participation and signed informed consent forms.

The study population included individuals between 30 and 100 years of age, among them 40 healthy probands, 17 patients diagnosed with early knee OA, and 148 patients diagnosed with late knee OA requiring TKA. Healthy probands were defined as persons without any prior knee joint injury, any diagnosed OA, and any joint pain or swelling. Early OA patients were defined as persons receiving OA diagnosis maximum 6 months before analysis and a maximum Kellgren-Lawrence (KL) score 2 and/or who suffered from a knee joint injury at least 6 months before analysis, and who have felt knee joint pain for maximum 4 months before analysis [26]. Late OA patients with KL > 2 scores scheduled for TKA were analyzed 1–4 days prior to surgery. Exclusion criteria for all groups comprised lower limb injuries, any lower limb surgeries within 6 months prior to study participation, former endoprosthetic surgeries of the hip or ankle joint, rheumatoid arthritis, or various neurological disorders such as Alzheimer's disease, cerebral palsy, depression, epilepsy, Huntington’s disease, multiple sclerosis, muscular dystrophy, paraneoplastic syndrome, Parkinson’s disease, poliomyelitis, stroke with paralysis, and tremor. Study participants’ data included sex, age, BMI, smoking status (currently smoking, ex-smoking, non-smoking), and medication. We collected relevant details of medication known to influence HRV (e.g. pain killers [7]). Study participant baseline characteristics are displayed in Table 1.

**Table 1:** Baseline characteristics of study participants

Characteristics	Healthy	Early OA	Late OA
Number	40	17	148
Women/ men, n (%)	24 (60)/16 (40)	8 (47)/9 (53)	80 (54)/68 (46)
Mean age (range) [yr]	53 (30–83)	49 (30–66)	66 (39–91)***
Mean BMI (range) [kg/m^2^]	26 (19–42)	26 (21–32)	32 (19–51)^###^
Non-smoker/smoker/ex-smoker, n (%)	30 (75)/7 (17.5)/0 (0)	12 (70.6)/4 (23.5)/1 (5.9)	78 (70)/28 (16)/6 (5)
Medication			
Painkiller, n (%)	0	1 (6)	66 (45)^###^
NSAIDs, n (%)	1 (3)	0	2 (1)
Alpha-/beta-blocker, n (%)	4 (10)	2 (12)	50 (34)
ARBs/ACE-inhibitors, n (%)	8 (20)	2 (12)	81 (55)^##^
Antidepressants/sedatives, n (%)	3 (8)	1 (6)	18 (12)

*ACE* angiotensin-converting-enzyme, *ARBs* angiotensin II receptor blockers, *NSAIDs* non-steroidal anti-inflammatory drugs, *yr* years

^***^p < 0.001 against healthy, Equal Variance Test (Brown-Forsythe), followed by Tukey Test; ^##^p < 0.01, ^###^p < 0.001 against healthy, Kruskal–Wallis One Way Analysis of Variance on Ranks, followed by Dunn’s Method

### Heart rate variability measurement

To quantify heart rate variability (HRV) in early and late OA patients, as well as healthy controls, we assessed time-domain HRV indices: SDRR (standard deviation of the detrended RR intervals), RMSSD (root mean square of successive RR interval differences), pRR50 (percentage of successive RR intervals that differ by more than 50 ms). We also assessed frequency-domain HRV indices: LF (absolute power of the low-frequency band (0.04–0.15 Hz)), HF (absolute power of the high-frequency band (0.15–0.4 Hz)), and the ratio LF/HF. While the LF band is widely recognized as an indicator of SNS activity, the HF band is often employed to reflect PNS activity. However, it is important to note that this is a simplified model, since LF and HF power are not pure indices of SNS and PNS drive, but also produced by other unspecified factors [27].

Electrocardiogram (ECG) rhythms were recorded using the AliveCor^®^ KardiaMobile System (KardiaMobile 6L) and associated FDA-certified App "Kardia". ECG rhythms were recorded for 5 min [17] between 9:30 to 11:30 am to avoid circadian influences, while patients were seated upright with their feet flat on the floor, limbs uncrossed, remaining still. The physiological data analysis software LabChart 8 Pro (AD Instruments) was used to calculate time-domain and frequency-domain HRV indices.

### Perceived stress and pain questionnaires

The perceived chronic stress level of the study participants was assessed using the German Perceived Stress Questionnaire-20 (PSQ-20) [28]. The PSQ-20 comprised 20 items referring different aspects of stress (worries, tension, joy, demands). Each item was rated on a Likert scale, ranging from 1 (hardly ever) to 4 (usually). The total score reflected the perceived chronic stress level of the respondent.

The perceived pain was assessed via Western Ontario and McMaster Universities Osteoarthritis Index (WOMAC 3.1) [29]. This questionnaire comprised 24 items referring pain severity, joint stiffness, and difficulties in performing daily activities. Each item was rated on a 11-point numerical rating scale, ranging from 0 (no pain/ stiffness/ difficulty) to 10 (extreme pain/ stiffness/ difficulty).

### Serum stress biomarker analysis

To assess SNS activity, serum concentration of the catecholamines dopamine (DA), norepinephrine (NE) and epinephrine (E) was determined via LC–MS/MS by the MVZ Dr. Eberhard & Partner Dortmund GbR, Germany. In addition, serum concentration of the HPA axis-related stress hormones cortisol and DHEA-S was determined via ELISA by the Institute for Clinical Chemistry, University Hospital Cologne, Germany.

### Data analysis

We initially included 40 healthy controls, 17 early OA patients and 155 late OA patients in the studying resulting in an overall sample size of 212 participants. Before data analysis, outlier were identified using the z-score method with exclusion for z >  + 2 and z < − 2 and removed from the data set. Outliers were identified via following formula: z-score = (value-mean)/standard deviation; outliers = z-score < 2. In total, 7 study participants were identified as outlier and therefore excluded from the subsequent analyses. After removal of outliers the data set used for analysis included 40 healthy controls, 17 early OA patients, and 148 late OA patients (n = 205 in total). The sample size indicated in the figure legends refers to the sample size used for the analysis after the removal of outliers. Linear regression analysis was performed with R (R Core Team (2022). R: A language and environment for statistical computing. R Foundation for Statistical Computing, Vienna, Austria) to identify the impact of grade of disease (GoD) (healthy, early OA, late OA) on heart rate variability (HRV), PSQ-20 and WOMAC pain scores, respectively. To elucidate possible confounders, we also adjusted the linear regression model for following baseline characteristics: female sex, age, BMI, CRP serum concentration, smoking behavior, intake of beta blockers, and pain medication. The impact of GoD as well as the impact of the covariants was determined via Kruskal–Wallis test, followed by post-hoc Dunn’s test for multiple comparison. Notably, via the linear regression analysis, we tested whether each estimate associated with GoD was significantly different from zero.

The box plots were generated using SigmaPlot (Systat Software, Erkrath, Germany) and display the median, the first and third quartiles, as well as outliers in the data. Inter-group differences of HRV parameters, PSQ, WOMAC, as well as serum parameters were analyzed via ANOVA on Ranks, followed by post-hoc Dunn’s test for multiple comparison. Quantile–quantile plots (q–q plots) were generated using the ggplot2 package (v.3.4.3; [30]) in RStudio (Posit Software, PBC) and visualize the distribution of the variables. Only individuals with a normal sinus rhythm were considered for the q–q-plots. p values less than 0.05 were considered significant.

Invariant correlations between HRV and PSQ or WOMAC and correlation between serum stress biomarkers and GoD, PSQ, or WOMAC were determined by assessing the Spearman’s rank correlation coefficient (R_s_) andrespective p-values were obtained using Package rstatix version 0.7.2 and heatmaps were created in R with the ggplot2 package version 3.5.0. Correlations are represented as heat map matrices as well as scatter plots, which were generated in R and SigmaPlot, respectively.

## Results

### Late OA patients demonstrated increased heart rate

**Fig. 1:**
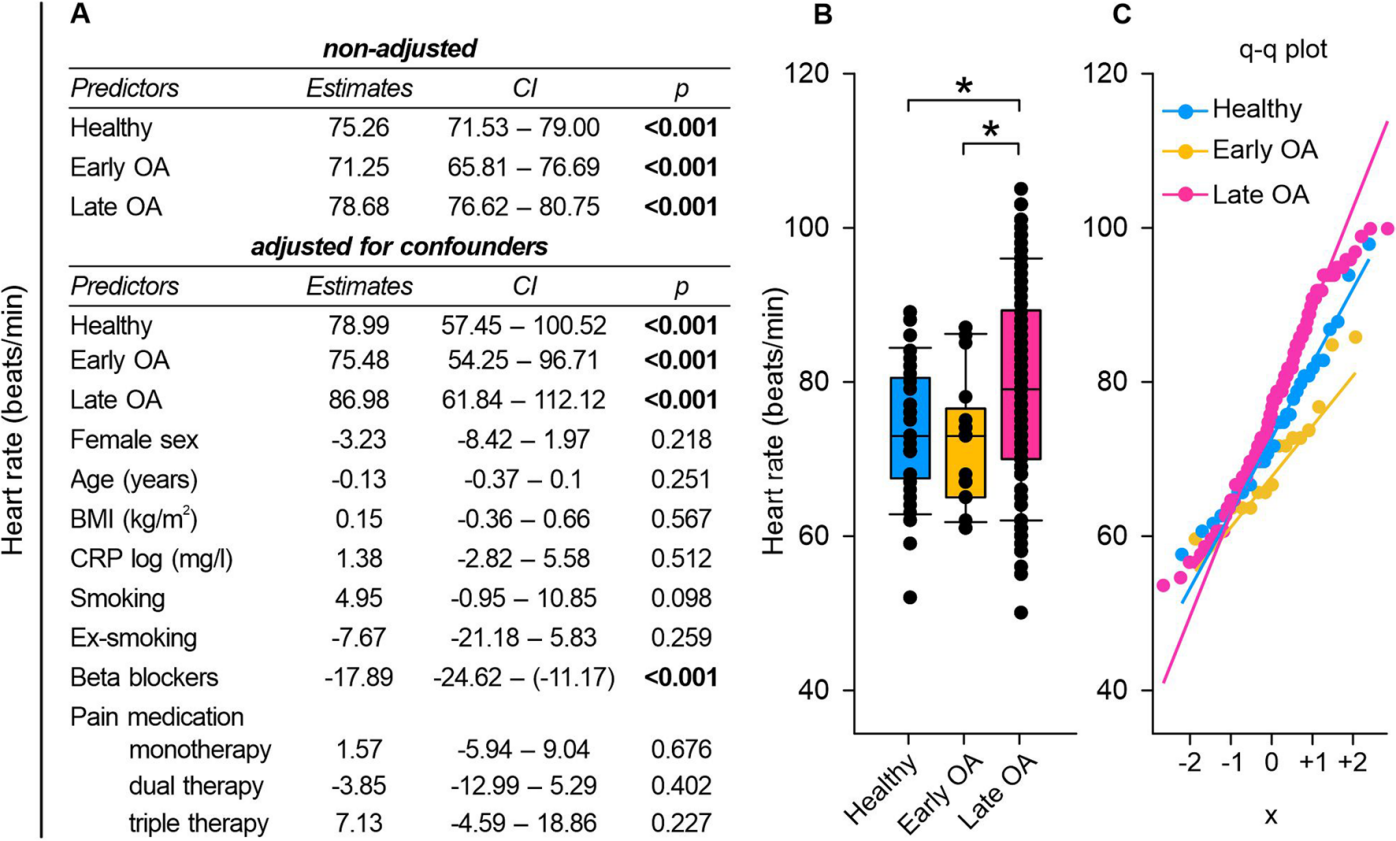
HR in healthy controls, early and late OA patients.** A** The impact of GoD on HR was analyzed via linear regression analysis. The parameters were generated separately, either in a non-adjusted model or a model adjusted for confounders. **B** HR of healthy controls, early OA patients, and late OA patients. Data are represented as box plot, where the boxes represent the 25th to 75th percentiles, the lines within the boxes represent the median, and the lines outside the boxes represent the 10th and 90th percentiles. Each circle represents an individual patient. **C** The q–q plot visualizes the distribution of HR in healthy controls, early OA patients and late OA patients. The x axis shows the normal theoretical quantiles. Only individuals with a normal sinus rhythm were considered for the q–q plots. Each circle displays an individual patient. Significant p-values are presented as *p ≤ 0.05. n (healthy) = 40, n (early OA) = 17, n (late OA) = 148. *HR* heart rate, *GoD* grade of disease, *CI* confidence interval, *q–q plot *quantile–quantile plot

Gaining a holistic perspective on the overall health of OA patients requires a comprehensive grasp of the disease effects on various body systems. One approach to contribute to this broader comprehension is the examination of heart rate (HR). The ECG analyses revealed distinct HR alterations in late OA patients. Late OA patients demonstrated a significantly elevated HR compared to healthy individuals and early OA patients (healthy vs. early OA p = 1.000, healthy vs. late OA p = 0.049, early OA vs. late OA p = 0.048) (Fig. 1).

### Time-domain HRV measurements: HRV decreased with OA severity

**Fig. 2:**
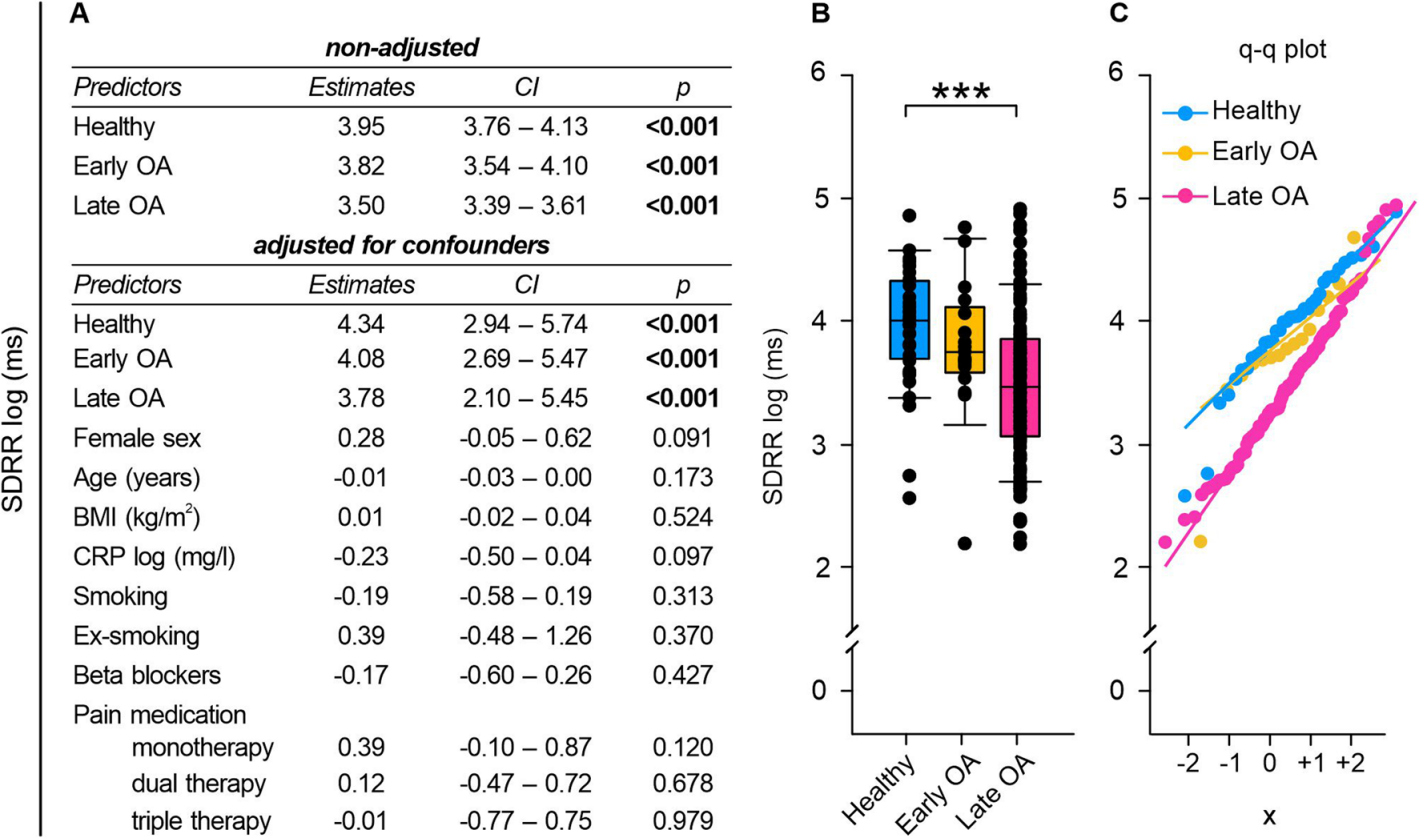
SDRR of healthy controls, early and late OA patients. **A** The impact of GoD on SDRR was analyzed via linear regression analysis. The parameters were generated in a non-adjusted model and a model adjusted for confounders, separately. **B** SDRR data of healthy controls, early OA patients, and late OA patients are represented as box plots, where the boxes represent the 25th to 75th percentiles, the lines within the boxes represent the median, and the lines outside the boxes represent the 10th and 90th percentiles. Each circle represents an individual patient. **C** The q–q plot visualizes the distribution of SDRR in healthy controls, early OA patients and late OA patients. The x axis shows the normal theoretical quantiles. Only individuals with a normal sinus rhythm were considered for the q–q plots. Each circle displays an individual patient. Significant p-values are presented as ***p ≤ 0.001. n (healthy) = 40, n (early OA) = 17, n (late OA) = 148. *GoD* grade of disease, *CI* confidence interval, *q–q plot *quantile-quantile plot

**Fig. 3:**
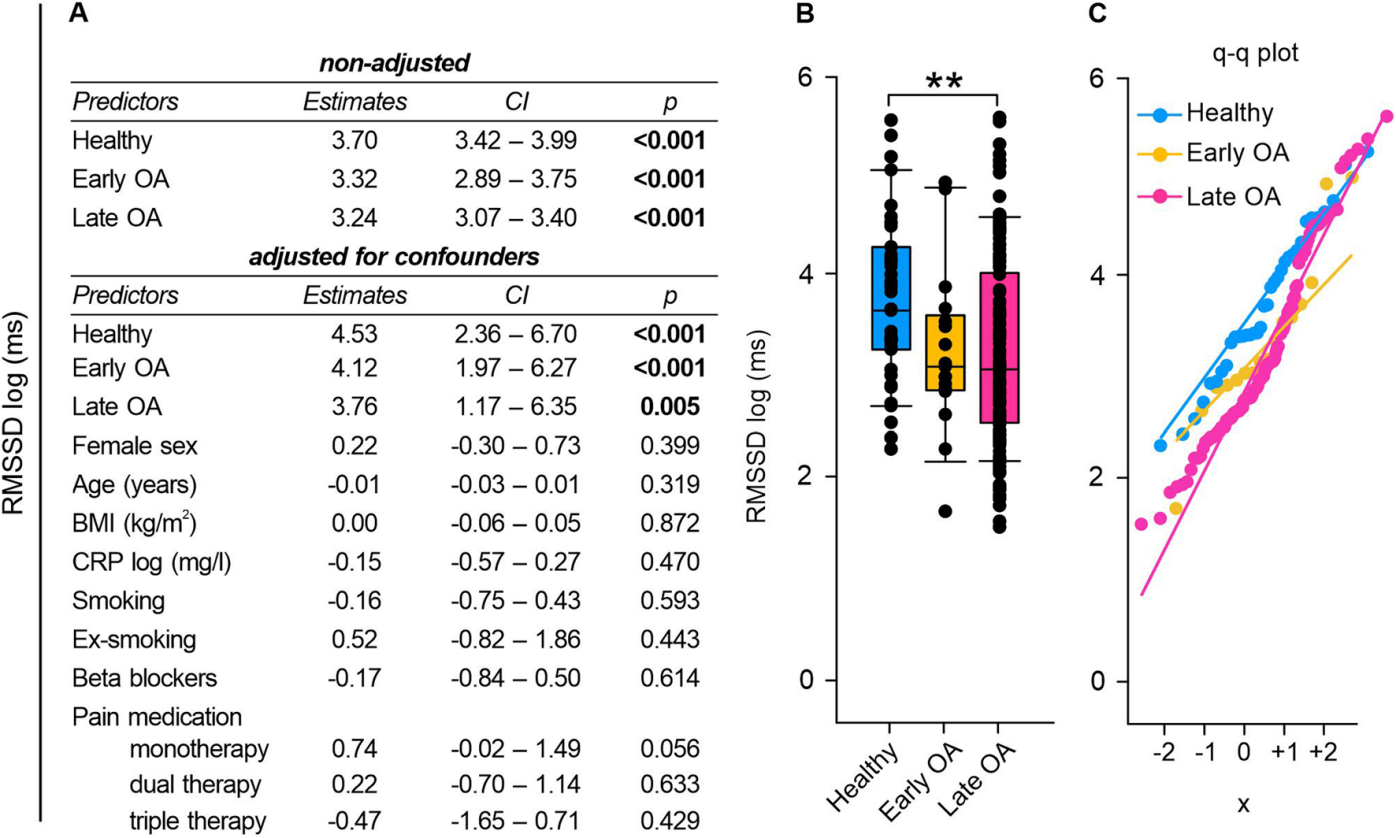
RMSSD of healthy controls, early and late OA patients. **A** The impact of GoD on RMSSD was analyzed via linear regression analysis. The parameters were generated in a non-adjusted model and a model adjusted for confounders, separately. **B** RMSSD data of healthy controls, early OA patients, and late OA patients are represented as box plots, where the boxes represent the 25th to 75th percentiles, the lines within the boxes represent the median, and the lines outside the boxes represent the 10th and 90th percentiles. Each circle represents an individual patient. **C** The q–q plot visualizes the distribution of RMSSD in healthy controls, early OA patients and late OA patients. The x axis shows the normal theoretical quantiles. Only individuals with a normal sinus rhythm were considered for the q–q plots. Each circle displays an individual patient. Significant p-values are presented as **p ≤ 0.01. n (healthy) = 40, n (early OA) = 17, n (late OA) = 148. *GoD* grade of disease, *CI* confidence interval, *q–q plot* quantile–quantile plot

**Fig. 4:**
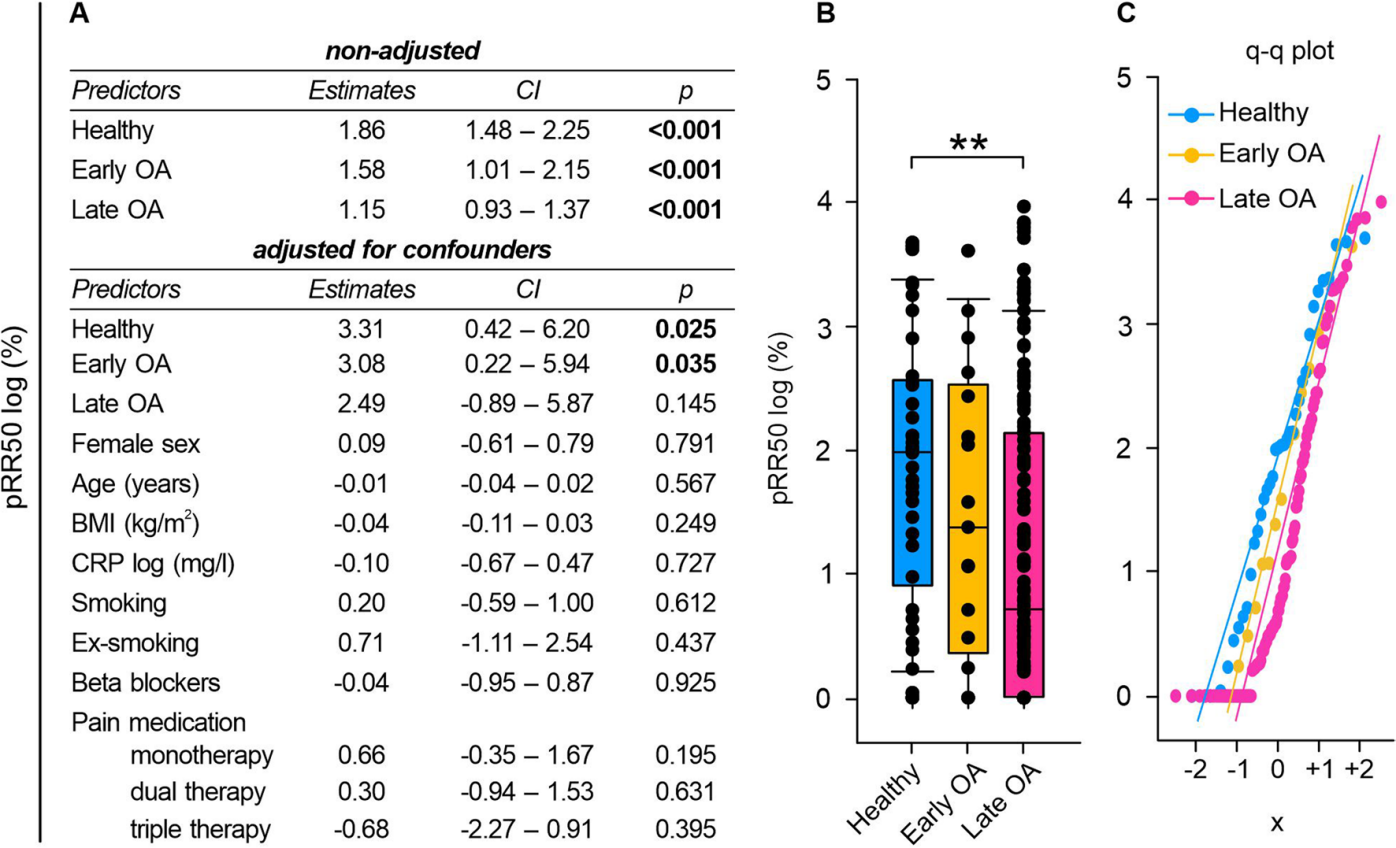
pRR50 of healthy controls, early and late OA patients. **A** The impact of GoD on pRR50 was analyzed via linear regression analysis. The parameters were generated in a non-adjusted model and a model adjusted for confounders, separately. **B** pRR50 data of healthy controls, early OA patients, and late OA patients are represented as box plots, where the boxes represent the 25th to 75th percentiles, the lines within the boxes represent the median, and the lines outside the boxes represent the 10th and 90th percentiles. Each circle represents an individual patient. **C** The q–q plot visualizes the distribution of pRR50 in healthy controls, early OA patients and late OA patients. The x axis shows the normal theoretical quantiles. Only individuals with a normal sinus rhythm were considered for the q–q plots. Each circle displays an individual patient. Significant p-values are presented as **p ≤ 0.01. n (healthy) = 40, n (early OA) = 17, n (late OA) = 148. *GoD* grade of disease, *CI* confidence interval, *q–q plot* quantile–quantile plot

Since HR is controlled by the ANS, we further investigated the autonomic function of OA patients via HRV analysis. Linear regression analysis revealed that GoD significantly impacted all time-domain HRV metrics (Figs. 2A, 3A, 4A). We subsequently adjusted our linear regression model to account for potential contribution from clinical baseline characteristics to this correlation. However, GoD remained the only significant contributor to changes in SDRR and RMSSD despite controlling for sex, age, BMI, CRP serum concentrations, smoking, beta blockers, and pain medication (Figs. 2A, 3A). After adjusting for potential confounders, only the status of healthy or early OA remained significantly associated with pRR50 (Fig. 4A). It is noteworthy, though, that less than 1% of successive RR intervals in late OA patients exhibited variations exceeding 50 ms on average (Fig. 4B).

The numeric values of SDRR, RMSSD, and pRR50 were significantly lower in late OA patients compared to healthy controls (SDRR: healthy vs. early OA p = 0.850, healthy vs. late OA p < 0.001, early OA vs. late OA p = 0.089; RMSSD: healthy vs. early OA p = 0.246, healthy vs. late OA p = 0.004, early OA vs. late OA p = 1.000); pRR50: healthy vs. early OA p = 0.890, healthy vs. late OA p = 0.004, early OA vs. late OA p = 0.771) (Figs. 2B, 3B, 4B).

However, some late OA patients exhibited remarkably high HRV values, on par with individuals demonstrating high HRV values from the group of healthy controls or early OA patients. Q–q plots displayed that discrepancies between groups were most pronounced in the negative quartiles, which might indicate that individuals with HRV below the mean showcased more pronounced inter-group differences than those with HRV values exceeding the mean (Figs. 2C, 3C, 4C).

### Frequency-domain HRV measurements: Early and late OA patients exhibited altered LF and HF power

**Fig. 5:**
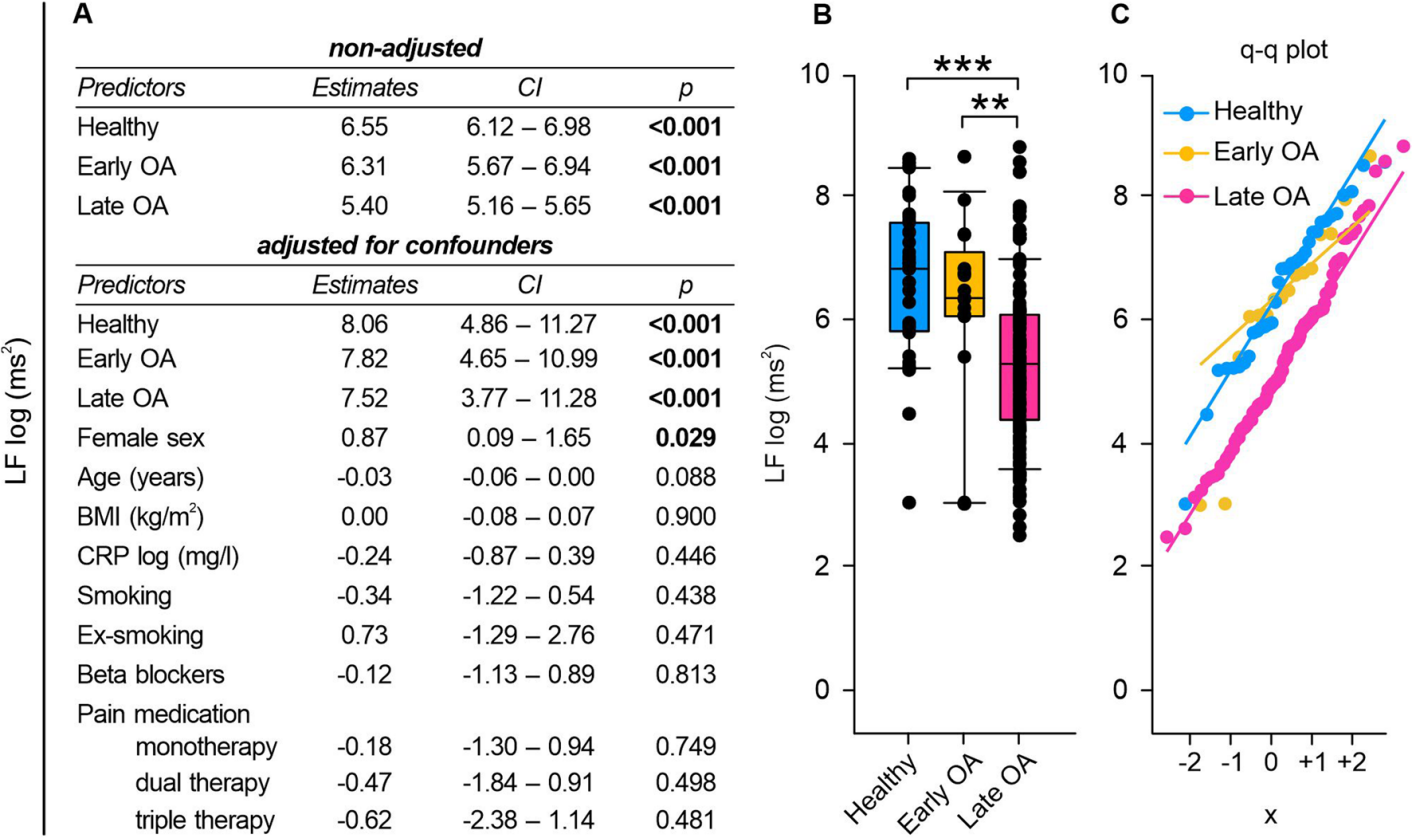
LF of healthy controls, early and late OA patients. **A** The impact of GoD on LF was analyzed via linear regression analysis. The parameters were generated in a non-adjusted model and a model adjusted for confounders, separately. **B** LF data of healthy controls, early OA patients, and late OA patients are represented as box plots, where the boxes represent the 25th to 75th percentiles, the lines within the boxes represent the median, and the lines outside the boxes represent the 10th and 90th percentiles. Each circle represents an individual patient. **C** The q–q plots visualize the distribution of LF in healthy controls, early OA patients and late OA patients. The x axis shows the normal theoretical quantiles. Only individuals with a normal sinus rhythm were considered for the q–q plots. Each circle displays an individual patient. Significant p-values are presented as **p ≤ 0.01, ***p ≤ 0.001. n (healthy) = 40, n (early OA) = 17, n (late OA) = 148. *GoD* grade of disease, *CI* confidence interval, *q–q plot* quantile–quantile plot

**Fig. 6:**
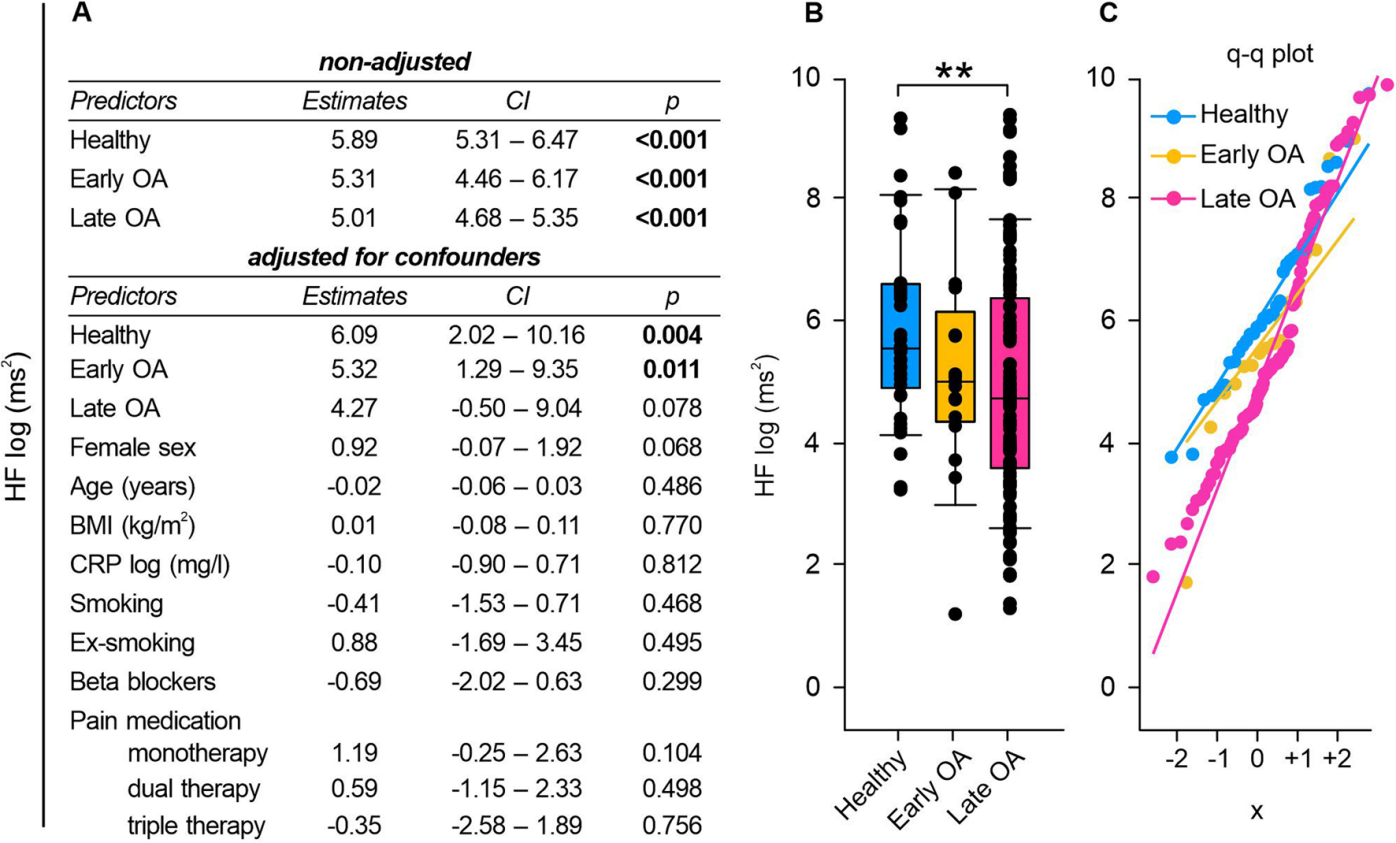
HF of healthy controls, early and late OA patients. **A** The impact of GoD on HF was analyzed via linear regression analysis. The parameters were generated in a non-adjusted model and a model adjusted for confounders, separately. **B** HF data of healthy controls, early OA patients, and late OA patients are represented as box plots, where the boxes represent the 25th to 75th percentiles, the lines within the boxes represent the median, and the lines outside the boxes represent the 10th and 90th percentiles. Each circle represents an individual patient. **C** The q–q plots visualize the distribution of HF in healthy controls, early OA patients and late OA patients. The x axis shows the normal theoretical quantiles. Only individuals with a normal sinus rhythm were considered for the q–q plots. Each circle displays an individual patient. Significant p-values are presented as **p ≤ 0.01. n (healthy) = 40, n (early OA) = 17, n (late OA) = 148. *GoD* grade of disease, *CI* confidence interval, *q–q plot* quantile–quantile plot

**Fig. 7:**
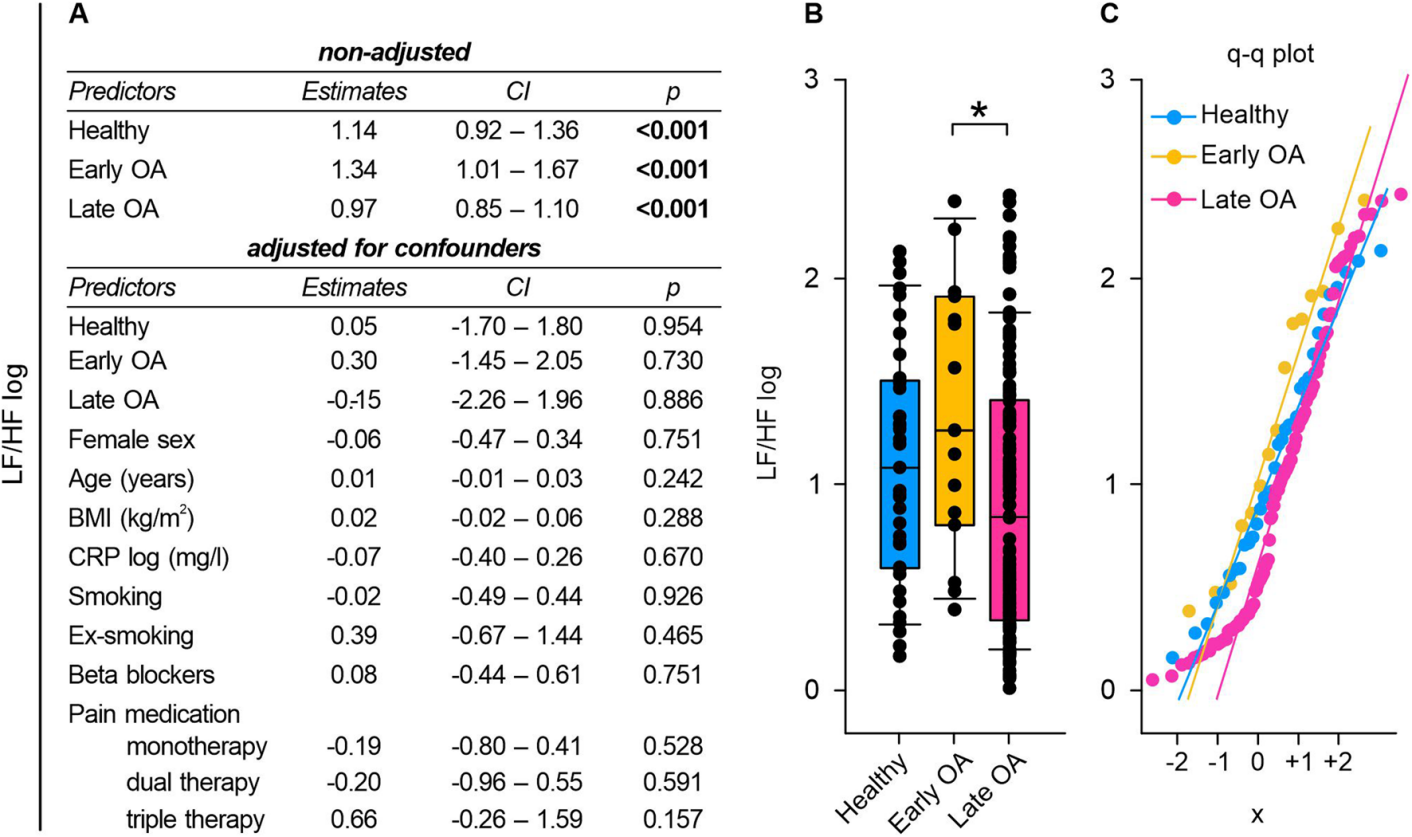
LF/HF of healthy controls, early and late OA patients. **A** The impact of GoD on LF/HF was analyzed via linear regression analysis. The parameters were generated in a non-adjusted model and a model adjusted for confounders, separately. **B** LF/HF data of healthy controls, early OA patients, and late OA patients are represented as box plots, where the boxes represent the 25th to 75th percentiles, the lines within the boxes represent the median, and the lines outside the boxes represent the 10th and 90th percentiles. Each circle represents an individual patient. **C** The q–q plots visualize the distribution of LF/HF in healthy controls, early OA patients and late OA patients. The x axis shows the normal theoretical quantiles. Only individuals with a normal sinus rhythm were considered for the q–q plots. Each circle displays an individual patient. Significant p-values are presented as *p ≤ 0.05. n (healthy) = 40, n (early OA) = 17, n (late OA) = 148. *GoD* grade of disease, *CI* confidence interval, *q–q plot *quantile–quantile plot

According to the non-adjusted regression analysis, LF and HF power as well as LF/HF ratio were significantly influenced by GoD (Figs. 5A, 6A, 7A). Following adjustments for confounding factors, only healthy and early OA status maintained a significant relationship with HF power (Fig. 6A). In the adjusted regression model, LF/HF ratio showed no significant association with the GoD (Fig. 7A).

Healthy subjects exhibited significantly higher LF and HF power compared to the LF and HF power seen in late OA patients (LF: healthy vs. early OA p = 0.347, healthy vs. late OA p < 0.001, early OA vs. late OA p = 0.004; HF: healthy vs. early OA p = 0.608, healthy vs. late OA p = 0.004, early OA vs. late OA p = 1.000) (Figs. 5B, 6B). Regression model adjustments revealed that female sex significantly augmented LF power (Fig. 5A).

Early OA patients presented a physiologically relevant but mathematically not significant increase in LF/HF compared to the control group, whereas late OA patients exhibited slightly lower LF/HF than the controls (healthy vs. early OA p = 0.827, healthy vs. late OA p = 0.234). Early OA patients demonstrated significantly higher LF/HF than late OA patients (early OA vs late OA p = 0.046) (Fig. 7B).

### Early and late OA patients perceived elevated chronic stress

**Fig. 8:**
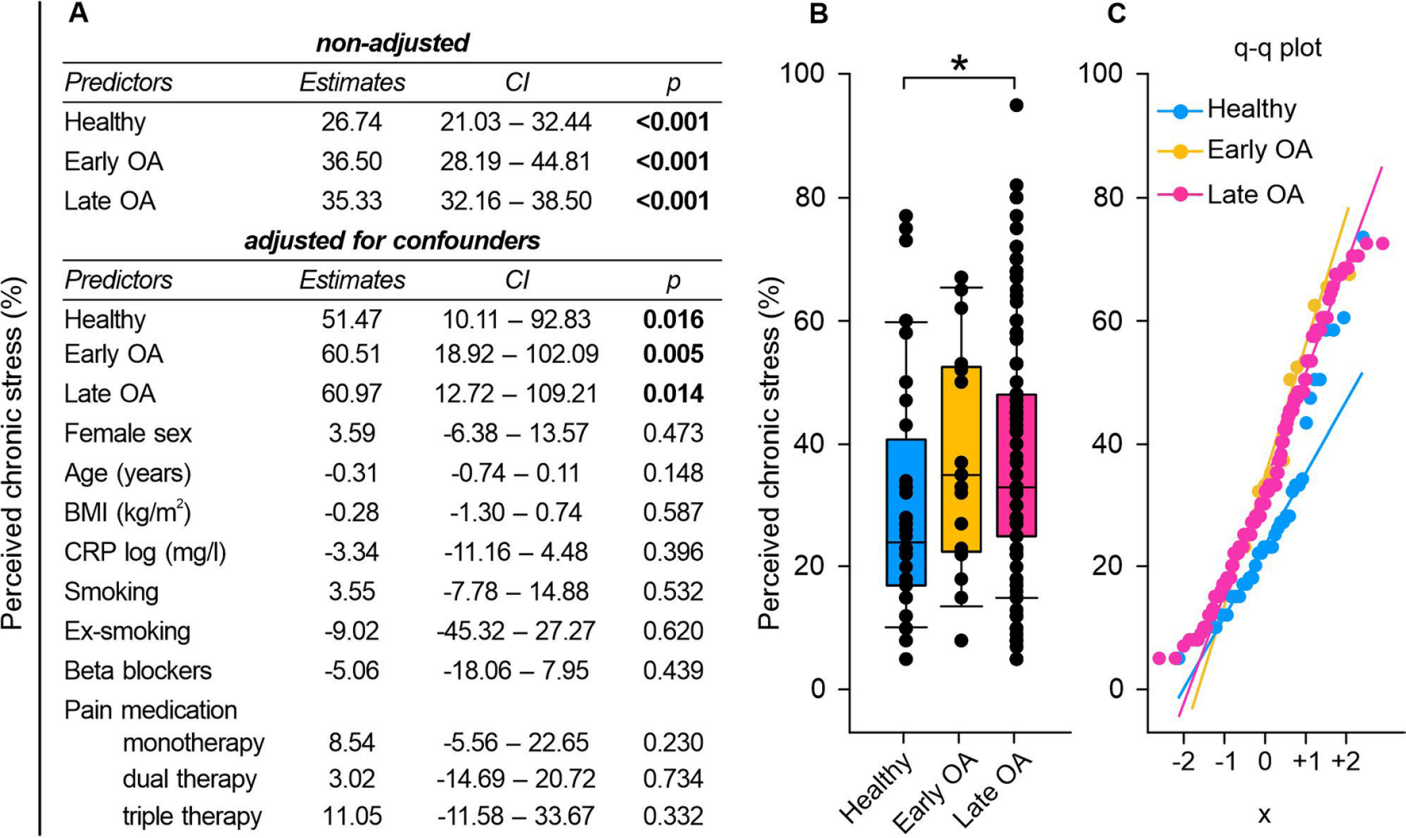
Perceived chronic stress of healthy controls, early and late OA patients. **A** The impact of GoD on perceived chronic stress was analyzed via linear regression analysis. The parameters were generated in a non-adjusted model and a model adjusted for confounders, separately. **B** Perceived chronic stress data of healthy controls, early OA patients, and late OA patients are represented as box plots, where the boxes represent the 25th to 75th percentiles, the lines within the boxes represent the median, and the lines outside the boxes represent the 10th and 90th percentiles. Each circle represents an individual patient. **C** The q–q plots visualize the distribution of perceived chronic stress in healthy controls, early OA patients and late OA patients. The x axis shows the normal theoretical quantiles. Only individuals with a normal sinus rhythm were considered for the q–q plots. Each circle displays an individual patient. Significant p-values are presented as *p ≤ 0.05. n (healthy) = 40, n (early OA) = 17, n (late OA) = 148. *GoD* grade of disease, *CI* confidence interval, *q–q plot* quantile–quantile plot

Since disease associated psychological alterations might be a contributor to HRV changes we investigated perceived stress level dependent on GoD [19]. In both non-adjusted and adjusted regression models GoD significantly impacted perceived chronic stress levels (Fig. 8A). Healthy individuals demonstrated significantly lower stress levels compared to late, but not early OA patients (healthy vs. early OA p = 0.312, healthy vs. late OA p = 0.031). Notably, the mean chronic stress level exhibited minimal variation between early and late OA patients (early OA vs. late OA p = 1.000) (Fig. 8B). Q–q plots revealed that differences between control and OA groups primarily occurred within the − 1 to + 1 quantile range. Despite mean stress values diverging between these groups, particularly low (− 2 quantile) and high (+ 2 quantile) values showed consistency across all groups. This indicates that every investigated group has members with very low and very high perceived stress not further altered by GoD (Fig. 8C). Correlation analysis revealed only week negative correlations between perceived chronic stress and HRV (Fig. 10A and Fig. S1A).

### Monotherapeutic pain medication and a smoking influenced the WOMAC pain level

**Fig. 9:**
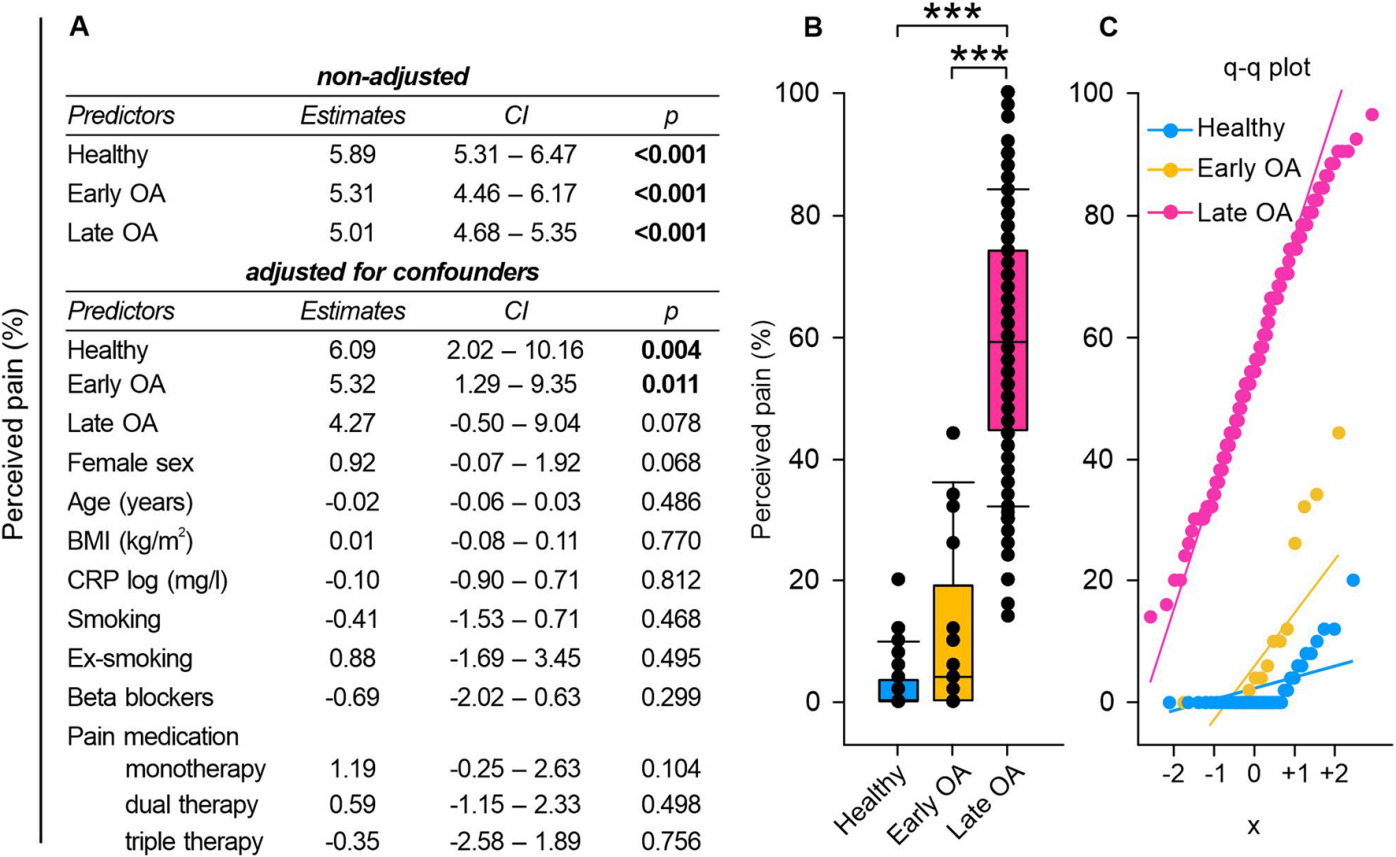
Perceived chronic pain of healthy controls, early and late OA patients. **A** The impact of GoD on perceived pain was analyzed via linear regression analysis. The parameters were generated in a non-adjusted model and a model adjusted for confounders, separately. **B** Perceived pain data of healthy controls, early OA patients, and late OA patients are represented as box plots, where the boxes represent the 25th to 75th percentiles, the lines within the boxes represent the median, and the lines outside the boxes represent the 10th and 90th percentiles. Each circle represents an individual patient. **C** The q–q plots visualize the distribution of perceived pain in healthy controls, early OA patients and late OA patients. The x axis shows the normal theoretical quantiles. Only individuals with a normal sinus rhythm were considered for the q–q plots. Each circle displays an individual patient. Significant p-values are presented as ***p ≤ 0.001. n (healthy) = 40, n (early OA) = 17, n (late OA) = 148. *GoD* grade of disease, *CI *confidence interval, *q–q plot *quantile–quantile plot

As the ANS is also involved in the subjective experience of pain [31], we further analyzed the perceived pain of the study participants. In the non-adjusted regression model, perceived pain levels were significantly influenced by both, early and late OA status. In the adjusted model, a significant impact on pain levels emerged exclusively for late OA patients (Fig. 9A). Adjusting for potential confounders indicated that monotherapeutic pain medication and a history of smoking significantly increased perceived pain (Fig. 9A). As expected, late OA patients perceived significantly more severe pain than early OA patients and healthy controls (healthy vs. early OA p = 1.000, healthy vs. late OA p < 0.001, early OA vs. late OA p < 0.001) (Fig. 9B).

**Fig. 10:**
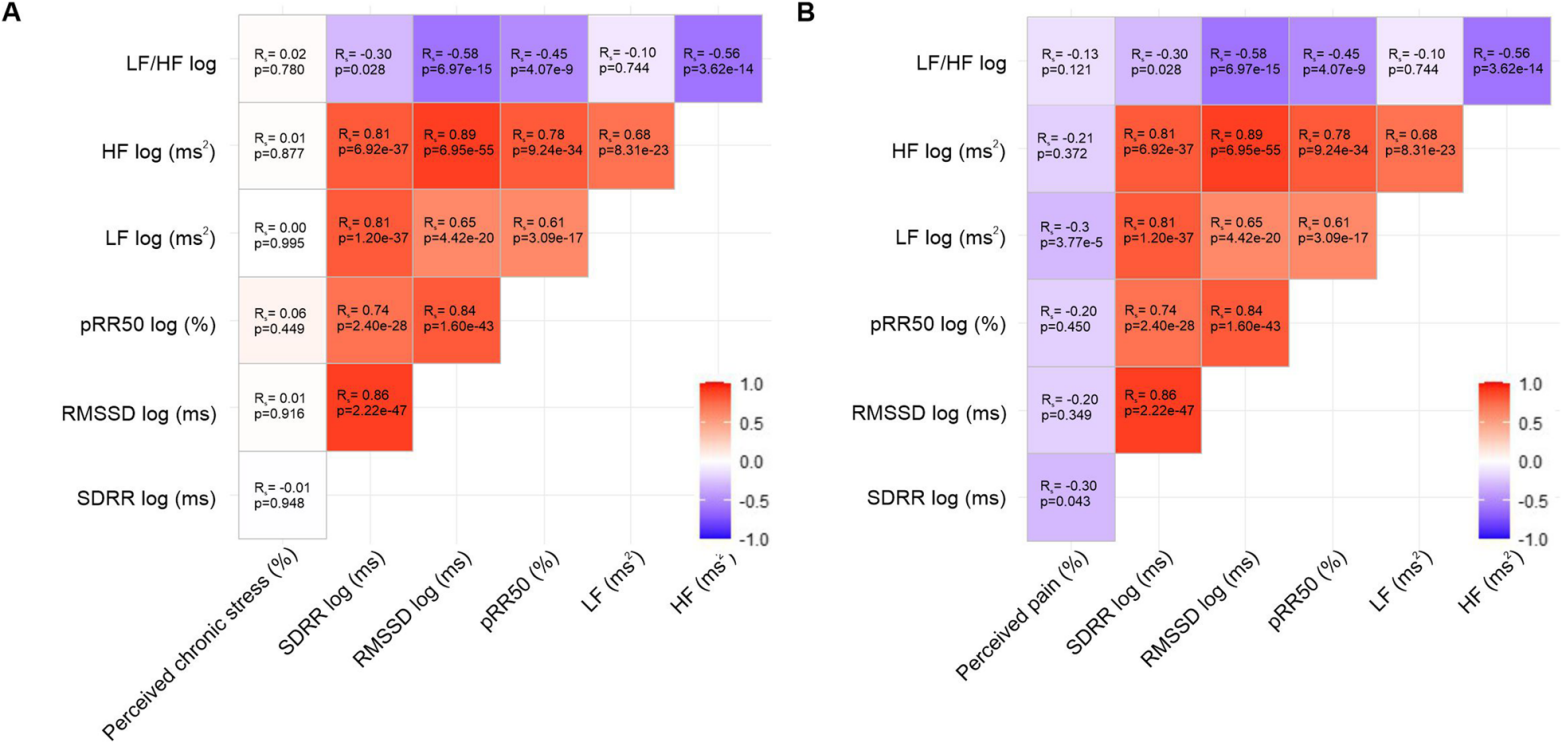
Correlations of HRV parameters with perceived chronic stress and chronic pain levels in healthy controls, early and late OA patients.** A** Correlation matrix (heat map) representing the relationships between HRV parameters and perceived chronic stress (n = 205). **B** Correlation matrix (heat map) representing the relationships between HRV parameters and perceived pain (n = 205). In each cell, correlation coefficients and respective p values are provided

Correlation analysis revealed only week negative correlations between perceived pain and HRV (Fig. 10B and Fig. S1B).

### Elevated serum stress hormone levels in late OA patients

**Fig. 11:**
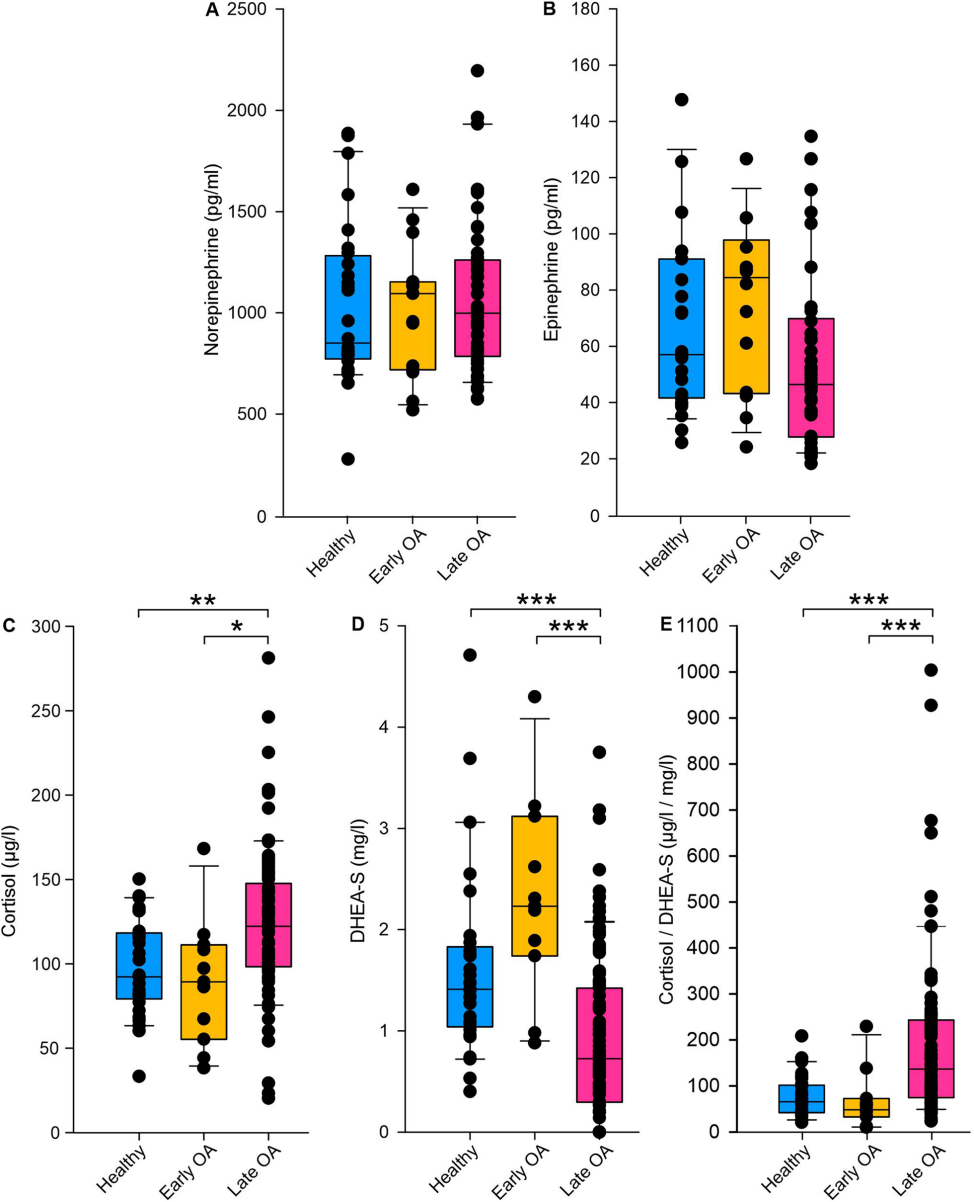
Stress-related serum biomarkers in healthy controls, early and late OA patients. Concentrations of **A** NE (healthy controls n = 28, early OA patients n = 15, late OA patients n = 49), **B** E (healthy controls n = 27, early OA patients n = 14, late OA patients n = 42), **C** cortisol (healthy controls n = 29, early OA patients n = 11, late OA patients n = 85), **D** DHEA-S (healthy controls n = 29, early OA patients n = 14, late OA patients n = 102), and **E** cortisol/DHEA-S (healthy controls n = 29, early OA patients n = 11, late OA patients n = 85) in the serum of study participants. Biomarker levels are represented as box plots with whiskers. Each circle represents an individual patient. Significant *p-*values between groups are presented as *p ≤ 0.05, **p ≤ 0.01, ***p ≤ 0.001. The black line represents the Spearman’s rank correlation with GoD

To asses sympathetic activity also at the molecular level, we quantified serum concentrations of the sympathetic neurotransmitters DA, NE and E. NE and E did not vary with GoD (NE: healthy vs. early OA p = 0,866, healthy vs. late OA p = 0,854, early OA vs. late OA p = 0,422; E: healthy vs. early OA p = 0.112, healthy vs. late OA p = 0.112, early OA vs. late OA p = 0.067) (Fig. 11A, B) and DA was not measurable in most samples (below detection limit). In contrast, the stress hormone cortisol concentration was significantly increased while the concentration of its counterpart DHEA-S was significantly reduced in late OA patients compared to healthy controls and early OA patients (cortisol: healthy vs. early OA p = 1.000, healthy vs. late OA p = 0.006, early OA vs. late OA p = 0.02; DHEA-S: healthy vs. early OA p = 0,338, healthy vs. late OA p < 0.001, early OA vs. late OA p < 0.001) (Fig. 11C, D). The ratio of cortisol/DHEA-S and was significantly higher in late OA patients compared to healthy controls and early OA patients (healthy vs. early OA p = 1.000, healthy vs. late OA p < 0.001, early OA vs. late OA p < 0.001) (Fig. 11E).

### WOMAC pain positively correlated with perceived chronic stress and serum stress hormone levels

**Fig. 12:**
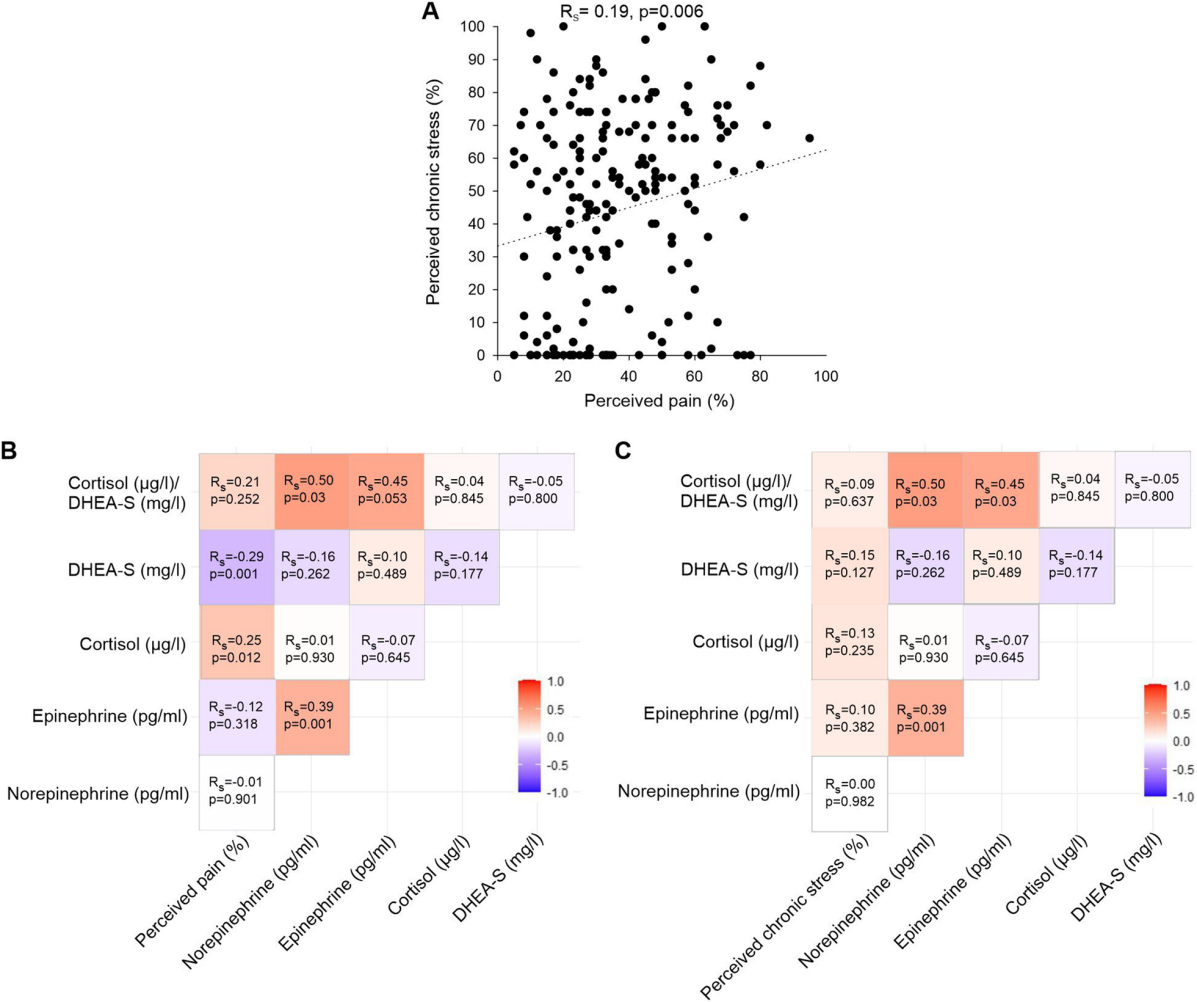
Correlations between perceived pain and chronic stress as well as stress-related serum biomarkers and perceived pain and chronic stress.** A** Correlation between perceived pain and chronic stress (n = 205). **B** Correlation matrix (heat map) representing the relationships between perceived pain and stress-related serum biomarkers. **C** Correlation matrix (heat map) representing the relationships between perceived chronic stress and stress-related serum biomarkers. For both correlation matrices n = 82 for E vs. perceived pain, perceived chronic stress, NE, cortisol, DHEA-S, cortisol/DHEA-S; n = 92 for NE vs. perceived pain, perceived chronic stress, cortisol, DHEA-S, cortisol/DHEA-S; n = 125 for cortisol vs. perceived pain, perceived chronic stress, DHEA-S, cortisol/DHEA-S; n = 125 for cortisol/DHEA-S vs. perceived pain, perceived chronic stress, DHEA-S; n = 145 for DHEA-S vs. perceived pain, perceived chronic stress. In each cell, correlation coefficients and respective p Values are provided

Perceived WOMAC pain positively correlated with perceived chronic stress (R_s_ = 0.19, very weak correlation; p = 0.01) (Fig. 12A). Moreover, WOMAC pain positively and significantly correlated with cortisol (R_s_ = 0.25, weak correlation; p = 0.00), while it negatively correlated with DHEA-S (R_s_ = − 0.29, weak correlation; p = 0.00) (Fig. S2B). Correlation analysis revealed a very weak correlation between NE and WOMAC pain, a weak correlation between E and WOMAC pain, as well as very weak correlations between biomarkers and PSQ (Fig. 12B, C and Fig. S2). Moreover, although NE and E concentrations did not significantly vary with GoD (Fig. 11A, B), the correlation between NE and cortisol/DHEA-S (R_s_ = 0.50, p = 0.03) was moderate and significant (Fig. 12B, C).

## Discussion

Despite the fact that OA is a widespread disease that has a significant impact on both individuals and society, there is still no causal treatment. To develop novel therapeutic approaches, it is critical to obtain a better understanding of this complex, multifactorial condition and its etiology also on a systemic level. Recent research suggests that OA patients may be prone to an autonomic dysfunction [24, 25], however, no study has systematically elucidated the autonomic tone in knee OA patients until now. To close this gap in knowledge, we conducted a prospective study with a large number of participants in order to make a well-founded statement about ANS involvement in OA. We collected cohorts of healthy probands as well as early and late OA patients with a comparable age range and considered numerous relevant baseline characteristics of the study participants to identify the most likely confounders of the analysis. Overall, our findings demonstrate that early and late knee OA patients seem to exhibit an autonomic dysfunction.

We started by examining HR because it provides an excellent summary of the patients' cardiovascular performance and indirectly also their general health status [32]. Moreover, elevated resting HR is associated with increased radiographic severity of knee OA [33]. In our study, late OA patients exhibited significantly higher HR values than healthy controls and early OA patients. Given that the ANS regulates HR [12], these results suggest that autonomic alterations, particularly in late OA, may exist. We further performed time-domain and frequency-domain ECG analysis to more deeply examine autonomic functions in OA.

Changes in HRV, indicated by time-domain indices RMSSD, SDRR, and pNN50, were significantly associated with GoD. Moreover, healthy controls demonstrated significantly higher numeric RMSSD, SDRR, and pNN55 values than late OA patients. Consequently, it can be assumed that GoD-related changes, which were observed in the linear regression analysis, may possibly originate from differences between the HRV in healthy controls and OA patients.

HRV is known to depend on individual characteristics, such as age and sex [34]. As HRV decreases by age [34], we included age as possible confounder in the adjusted regression model. However, age did not have a significant impact on HRV throughout the analysis. It seems, that the influence of GoD outpaced the influence of age. This may be because the average age of the participants was relatively high and the differences between the groups were rather small. Thus, the influence of age on HRV in this study is negligible. Similarly, it is not surprising that sex had no significant effect on HRV in our cohort with mean age ≥ 49 years as the sex differences in HRV are known to decline at age older than 30 years and eliminate at age over 50 years [34]. Some studies reported that also BMI [35] and smoking [36] are related to low HRV. In our study, these parameters did not influence time-domain HRV indices. This finding demonstrates that the observed impact of GoD on HRV is primarily due to the diagnosis and that influence of other tested factors is negligible. HRV is mainly regulated by the parasympathetic vagus nerve [37] and consequently, OA patients seem to have a PNS deficiency that already occurs in the early stage of the disease. It is well known that PNS activation via the cholinergic signaling pathway exhibits anti-inflammatory effects in OA [38]. For example, human OA synovium and synoviocytes express the alpha7 nicotinic acetyl receptor (α7nAChR) that inhibits cytokine expression upon activation [39]. Moreover, α7nAChR activation mitigated OA progression by inhibiting inflammasome activation in rats [40]. Parasympathetic activity also attenuates extracellular matrix degradation: MMP-9 expression was reduced in OA mice via α7nAChR signaling [41]. As these parasympathetic effects are OA protective, a PNS deficit could be a risk factor for OA. This hypothesis is supported by findings of Liu et al. demonstrating that α7nAChR stimulation prevented monosodium iodacetate-induced OA in rats [42].

However, it needs to be taken into account that HRV is not only regulated by PNS activity, but indirectly also by the SNS and additionally, influenced by other factors, such as respiration [43]. Therefore, we further measured frequency-domain HRV parameters. Although LF and HF power are not generated exclusively by the SNS and PNS, they are valid indices to classify systemic SNS and PNS activity [17]. When interpreting the LF/HF index, it should be noted that there is no linear interaction between sympathetic and parasympathetic activity [27]. Nevertheless, LF/HF is an established parameter to depict the balance of both systems [17]. In our study, significantly decreased HF power in ECG recordings of OA patients verified that PNS activity diminished in late OA. Moreover, measurement of LF power revealed, that also SNS activity significantly decreased with OA severity. Numeric values of LF/HF were significantly lower in late OA patients compared to the early stage of the disease. It may be that the decline in PNS activity occurs earlier or progresses faster than the sympathetic reduction. This may result in a shift towards a dominant SNS at the onset of the disease reflected by an increased LF/HF. It could be that the decrease in LF/HF in late OA is an attempt to compensate for the sympathetic dominance. An increased LF/HF ratio, as detected in early OA patients, has already been reported in RA patients [44]. Moreover, the SNS is known to promote mild chronic inflammation in OA [9]. The declined sympathetic and parasympathetic activity together with the occurring imbalance of both systems indicate an autonomic dysfunction in OA.

Previously, HRV abnormalities have been implicated in numerous psychopathologies (Heiss 2020). A decreased HRV is related to a lower ability to regulate emotions (reviewed in [45] and accordingly, also to stress [46]. As OA patients in our study seem to exhibit autonomic dysfunction, we investigated if they also experience chronic stress. Early- and late OA patients demonstrated increased elevated perceived chronic stress levels compared to healthy controls. In addition, late OA patients exhibited increased serum levels of stress-related cortisol and decreased concentrations of its counterpart DHEA-S. These results demonstrate an increased stress level also at the molecular level and thus confirm our HRV data [47]. Moreover, also an increased ratio of cortisol/DHEA-S indicated a shift towards a more catabolic status and increased chronic stress in OA patients [13].This is consistent with research from Ackermann et al. demonstrating that patients on the waiting list for knee or hip arthroplasty experience psychologic distress five times more frequently than the general population [48]. Consequently, it could be speculated that OA-related autonomic dysfunction may increase stress. This hypothesis is supported by findings from Rösch et al., reporting elevated concentrations of the sympathetic neurotransmitter and stress hormone NE eight weeks after surgical OA induction in splenic lysates of mice [49]. In contrast, in our study, NE and E levels did not vary significantly between healthy controls and OA patients. However, this could be due to a faster turnover rate of serum compared to splenic catecholamines [50]. Accordingly, Leinhard et al. stated that serum catecholamine levels might not be suitable to detect SNS activity in humans [51]. In the present study, early OA patients already perceived the same amount of chronic stress as patients in the late stage of the disease. Therefore, an autonomic dysfunction together with chronic stress could also be a predisposition for OA in humans. The second hypothesis is promoted by the sympathetic dominance detected in early OA patients. Moreover, Weber et al. described that low HRV levels are accompanied with a delayed recovery not only from psychological, but also immune stressors [52]. Therefore, individuals with low HRV could be prone to inflammatory diseases maybe increasing the risk for some inflammatory subtypes of OA. To clarify if the autonomic dysfunction is a risk factor for OA or rather a consequence of the disease, it would be useful to examine the autonomic tone in OA patients after TKA to determine if the autonomic dysfunction recovers.

According to Yeater et al. there might be a functional overlap between dysregulation of the autonomic and the pain regulation system [24]. In general, higher parasympathetic activity is associated with higher pain inhibition capacity [31]. As expected, the group of late OA patients demonstrated the highest pain level. Interestingly, the pain scores within this group exhibited a high variability. This reflects the common discrepancy between radiographic findings and pain in OA patients [53]. As the pain score raised mainly in late OA patients, it could not be the trigger for the above described high stress level in early OA patients.

As there is no curative treatment for OA, early detection and prevention of the disease is an urgent need. Our study elucidates that OA patients exhibit an autonomic dysfunction that already occurs in early patients. Consequently, time- and frequency-domain analysis could provide an easy, fast, non-invasive, and cheap way to detect the onset of OA. Especially since pain seems to occur only in the late stage of the disease. Vagus nerve stimulation (VNS) is currently investigated as anti-inflammatory therapeutic for treatment-resistant epilepsy, cardiovascular disease, rheumatoid arthritis, Crohn's disease, and asthma [54]. As inflammation also contributes to OA pathogenesis, this ANS-modulating treatment strategy may also be useful for OA therapy.

Courties et al. recently performed a pilot trial demonstrating that (VNS) may decrease clinical symptoms and joint inflammation in patients with erosive hand OA [38]. Moreover, non-invasive (VNS) also reduced sympathetic nerve activity in healthy humans [55]. Consequently, ANS-modulation by VNS may be useful not only to reduce inflammation by increasing parasympathetic activity but also to balance SNS and PNS and restore autonomic homeostasis.

Given the scope of our study, it is important to point out certain limitations. First, the participant cohort of both healthy individuals and early OA patients was relatively small in size, particularly in the early OA group. This constraint may have influenced the statistical power of our analysis, potentially impacting our ability to detect subtle differences. Moreover, inequalities across patient groups could have limited the applicability of our results. OA is known to be a heterogenous disease with many different phenotypes. However, we did not differentiate between different phenotypes within the patient cohort in the present study. Therefore, some effects may have been masked by the large variability of the data [56, 57]. This aspect will be addressed in future investigations using larger cohorts. We measured HRV using short-term ECG recordings, which might not accurately reflect the dynamic nature of autonomic activity as long-term HRV measures do. Additionally, it is essential to note that perceived stress and pain, pivotal outcome measures in our study, can be influenced by psychiatric comorbidities like depression, a factor that was not controlled for in our analysis. Furthermore, the observational nature of our study design inherently restricted our capacity to establish causal relationships between the variables under investigation. Therefore, while our findings suggest that several HRV parameters are associated with OA grade, the results do not provide compelling evidence that they are a risk factor in causal terms.

## Conclusion

In conclusion, our study has successfully illuminated the relevance of the autonomic tone in knee OA patients by measuring different well-established time- as well as frequency-domain HRV parameters. For the first time, OA patients indicate an autonomic dysfunction with indirect sympathetic dominance already at the early onset of the disease. These findings were substantiated by serum stress hormone analyses revealing that catecholamine and cortisol concentrations are influenced in the same way by the grade of OA. Furthermore, OA-related perceived pain and stress levels are potentially linked to this autonomic dysfunction. These findings become even more relevant when taking into account that an increased sympathetic activity and low-grade chronic inflammation reinforce each other in OA but also in OA comorbidities, such as hypertension, obesity, diabetes, and depression [9]. Consequently, these findings not only contribute to a more comprehensive understanding of the autonomic aspects of OA pathogenesis but also provide a link to the major OA comorbidities, shedding light on potential future therapeutic strategies targeting the ANS.

## Patient and public involvement

Patients and/or the public were not involved in the design, or conduct, or reporting, or dissemination plans of this research.

### Supplementary Information

Supplementary Figure 1. Correlation between perceived stress and HRV indices. A Correlation between perceived chronic stress and the HRV indices SDRR, RMSSD, pRR50, LF, HF, and LF/HF (n=205). B Correlation between WOMAC pain and the HRV indices SDRR, RMSSD, pRR50, LF, HF, and LF/HF (n=205). Each circle represents an individual patient. The dotted black line represents the Spearman’s rank correlation in assumption that data follow a linear correlation.

Supplementary Figure 2. Correlation between stress-related serum biomarkers and perceived stress or WOMAC pain. A Correlation between PSQ and the stress-related biomarkers NE (n=92), E (n=83), cortisol (n=125), DHEA-S (n=145), and cortisol/DHEA-S (n=125). B Correlation between WOMAC pain and the stress-related biomarkers NE (n=92), E (n=83), cortisol (n=125), DHEA-S (n=145), and cortisol/DHEA-S (n=125). Each circle represents an individual patient. The black dotted line represents the Spearman’s rank correlation in assumption that data follow a linear correlation.

## Data Availability

Data supporting the findings of this study are available from the corresponding author on reasonable request.

## Abbreviations

ACE: Angiotensin-converting-enzyme
ANS: Autonomic nervous system
ARBs: Angiotensin II receptor blockers
DA: Dopamine
DHEA-S: Dehydroepiandrosterone sulfate
E: Epinephrine
GoD: Grade of disease
HF: High frequency
HR: Heart rate
HRV: Heart rate variability
KL: Kellgren-Lawrence
LF: Low frequency
NE: Norepinephrine
NSAIDs: Non-steroidal anti-inflammatory drugs
OA: Osteoarthritis
PNS: Parasympathetic nervous system
PSQ: Perceived Stress Questionnaire-20
pRR50: Percentage of successive RR intervals that differ by more than 50 ms
RA: Rheumatoid arthritis
RMSSD: Root mean square of successive RR interval differences
SDRR: Standard deviation of the detrended RR intervals
SNS: Sympathetic nervous system
TKA: Total knee arthroplasty
WOMAC: Western Ontario and McMaster Universities Osteoarthritis Index
yr: Years
