# Supplementary figures and images for: Osteoarthritis patients exhibit an autonomic dysfunction with indirect sympathetic dominance
Source: J Transl Med. 2024 May 16;22:467. doi: 10.1186/s12967-024-05258-9 (PMC11100157; doi:10.1186/s12967-024-05258-9)

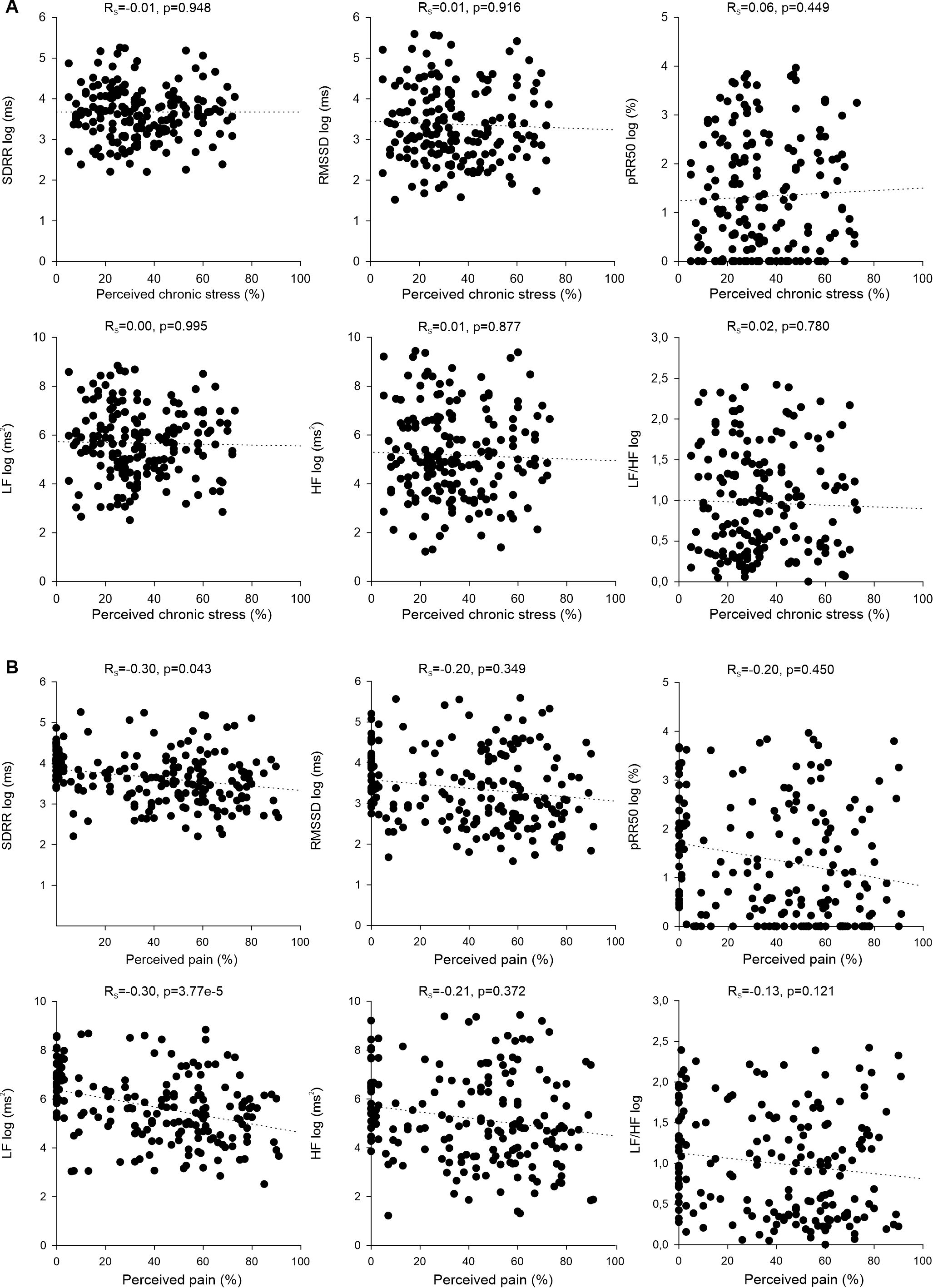

Supplement: Supplementary file 1 — Supplementary Figure 1. Correlation between perceived stress and HRV indices. A Correlation between perceived chronic stress and the HRV indices SDRR, RMSSD, pRR50, LF, HF, and LF/HF (n=205). B Correlation between WOMAC pain and the HRV indices SDRR, RMSSD, pRR50, LF, HF, and LF/HF (n=205). Each circle represents an individual patient. The dotted black line represents the Spearman’s rank correlation in assumption that data follow a linear correlation. [file 12967_2024_5258_MOESM1_ESM.jpg]

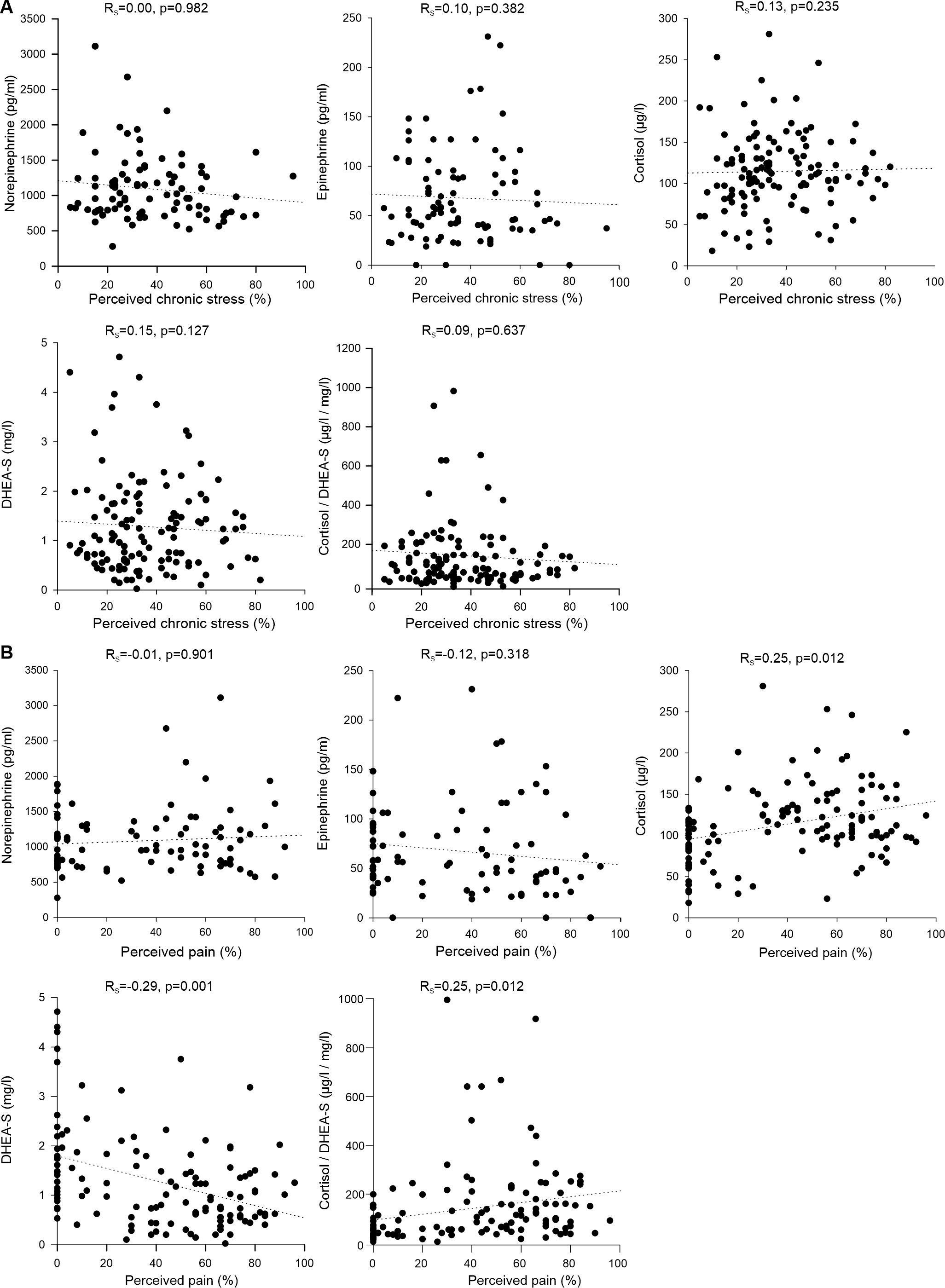

Supplement: Supplementary file 2 — Supplementary Figure 2. Correlation between stress-related serum biomarkers and perceived stress or WOMAC pain. A Correlation between PSQ and the stress-related biomarkers NE (n=92), E (n=83), cortisol (n=125), DHEA-S (n=145), and cortisol/DHEA-S (n=125). B Correlation between WOMAC pain and the stress-related biomarkers NE (n=92), E (n=83), cortisol (n=125), DHEA-S (n=145), and cortisol/DHEA-S (n=125). Each circle represents an individual patient. The black dotted line represents the Spearman’s rank correlation in assumption that data follow a linear correlation. [file 12967_2024_5258_MOESM2_ESM.jpg]
